# Intraspecific variation for heat stress tolerance in wild emmer-derived durum wheat populations

**DOI:** 10.3389/fpls.2025.1523562

**Published:** 2025-01-23

**Authors:** Mohammed Yousif Balla, Nasrein Mohamed Kamal, Izzat Sidahmed Ali Tahir, Yasir Serag Alnor Gorafi, Modather Galal Abdeldaim Abdalla, Hisashi Tsujimoto

**Affiliations:** ^1^ Arid Land Research Center, Tottori University, Tottori, Japan; ^2^ Wheat Research Program, Agricultural Research Corporation, Wad Medani, Sudan; ^3^ International Platform for Dryland Research and Education, Tottori University, Tottori, Japan; ^4^ Graduate School of Agriculture, Kyoto University, Kyoto, Japan

**Keywords:** heat resilient traits, wild emmer-derivative families, southern and northern lineages, drylands, diversity

## Abstract

High temperatures pose a major threat to wheat productivity and necessitate the development of new cultivars that are resilient to future heat stress. Wild emmer (*Triticum turgidum* L. ssp. *dicoccoides*), which is a direct progenitor of domesticated durum wheat (*Triticum turgidum* L. ssp. *durum*) and contributor to the A and B genome of bread wheat (*Triticum aestivum*), offers a valuable genetic reservoir for developing climate-resilient wheat. However, the morphology of wild emmer is different from that of durum and bread wheat, in particular, the spikelets are fragile and naturally fall off, making it difficult to study its agronomic traits. In this study, we created nine backcrossed families between the popular durum wheat cultivar ‘Miki 3’ and nine wild emmer accessions collected from northern and southern lineages of this species. The objective was to investigate the intraspecific genetic variation in wild emmer and identify traits associated with heat stress tolerance. We evaluated these nine families under multi-environments ranging from optimum to severe heat stress conditions in Japan and Sudan and measured important agronomic traits. The result showed that two families, developed from accessions of both northern and southern lineages exhibited high harvest index, elevated chlorophyll content, and reduced canopy temperature under heat stress. Additionally, one family developed from an accession of the southern lineage displayed high biomass, harvest index, and seed number under heat-stress conditions. These three families produced high heat tolerant lines with unique introgressed segments from their wild emmer parents on chromosomes 1A, 2B, 5B, 6B, and 7B, which may be linked to heat resilience. From these results, we were able to identify significant intraspecific diversity between the wild emmer accessions in terms of heat stress tolerance. However, no significant tendency between the northern and southern lineages of wild emmer has been identified. These findings emphasize the need to harness not only the interspecific but also the intraspecific genetic variation of wild emmer diversity to uncover valuable genes for heat stress tolerance in wheat breeding programs.

## Introduction

Wheat (*Triticum* spp.) is one of the cornerstones of global food security, with approximately 2.5 billion people relying on wheat products in their daily diet (Reynolds and Braun, 2022). However, the productivity of current wheat cultivars is increasingly limited by climate change scenarios, particularly due to rising temperatures (Narayanan, 2018; Iizumi et al., 2021). This limitation stems from narrow genetic diversity in modern wheat cultivars, a consequence of domestication events and successive breeding processes, restricting the potential for breeding heat-stress tolerant varieties. To broaden this diversity, utilizing genetic variation in wild wheat relatives offers a crucial resource for developing stress-resilient cultivars. For instance, in hexaploid wheat (*T*. *aestivum* L. genomes AABBDD), significant intraspecific diversity from *Aegilops tauschii* has been introduced into the D-genome of cultivated bread wheat (Tsujimoto et al., 2015), and the utility of this diversity has been demonstrated for improving tolerance to various stresses, including high temperatures (Elbashir et al., 2017; Gorafi et al., 2018; Elhadi et al., 2021a, 2021b; Itam et al., 2021; Mohamed et al., 2022). Similarly, in tetraploid wheat (*T*. *turgidum* L. ssp. *durum*, genomes AABB), the genetic diversity of wild emmer (*T*. *turgidum* L. ssp. *dicoccoides*), has been widely explored for enhancing stress resilience (Peng et al., 2013; El Haddad et al., 2021). This species occupies a wide altitude range, from 100 meters below to 1,800 meters above sea level, adapting to the very different climate conditions that vary from cool and humid slopes of the Karacadağ mountains to the hot and dry valleys in the North and South Fertile Crescent region, respectively (Özkan et al., 2011; Mastrangelo et al., 2023). Wild emmer, which shares the same A and B genome structure as cultivated bread and durum wheat, possesses key agronomic, physiological, and yield-related traits associated with stress tolerance (Peng et al., 2013). To harness this genetic potential, a set of wild emmer derivative (WED) families has been established in a durum wheat genetic background (Tsujimoto et al., 2015). The WED families combined genetic diversity from nine wild emmer accessions collected from the two main diverse lineages of natural distribution: the southern and northern lineages from the Southern Levant and Northern Levant, respectively, in the Fertile Crescent region (Ozkan et al., 2005; Peng et al., 2011). The molecular analysis of the WED families showed nine genetic groups, reflecting the genetic makeup of the nine wild emmer wheat accessions (Balla et al., 2022a). However, Balla et al. (2022b) evaluated the WED families as one population under the heat stress conditions in Sudan and found that the most heat stress tolerant lines were derived from different wild emmer parents. According to these promising results, we hypothesized that the allele contribution from the wild emmer offers multiple mechanisms in response to heat stress. These findings, coupled with the scarce studies on exploring the intraspecific variation of wheat wild relatives in the background of elite durum wheat, prompted the interest in studying the intraspecific diversity of wild emmer wheat by using the WED families to identify heat stress tolerance-associated traits that can be used in practical wheat breeding. Most previous studies on the intraspecific variation focused on evaluating the wheat wild relative species themselves. For instance, wild emmer has been explored for resistance to powdery mildew, leaf rust, and stripe rust resistance (Nevo et al., 1985), agronomic traits, protein content, and seed characters (Nevo et al., 1984), drought resistance and ecogeographical association (Peleg et al., 2005). Similarly, Mahjoob et al. (2022) evaluated 293 accessions of diploid wild wheat *Ae. tauschii* (the D-genome donor of hexaploid wheat) and explained the importance of intraspecific diversity and lineage differences regarding leaf hair density trait. In spite of the potential of the wheat wild relatives, including wild emmer, to contribute significantly to enhance the wheat genetic diversity, it has not been fully explored. This is mainly because the morphology of wild emmer differs from that of durum wheat and has many undesirable traits that make its direct evaluation difficult. In particular, the spikelets are fragile and naturally fall off, making the study of its agronomic traits difficult and potentially unreliable, and even the traits of the wild emmer may not be fully expressed at the tetraploid and hexaploid levels. This usually results in obscuring the genetic value and the exploration of useful alleles genes in the breeding programs. To overcome these obstacles, wild emmer-derived durum wheat populations were developed and proved to harbour useful climate-resilient traits derived from different wild emmer parents. However, the evaluation of intraspecific variation of wheat wild relatives on elite wheat genetic background under field conditions has yet to be fully explored.

This study aimed to elucidate the intraspecific genetic variation of wild emmer in the background of elite durum wheat under field-based heat stress conditions. Among the nine families, two families developed from accessions collected from southern and northern lineages showed a high harvest index, high chlorophyll content at maturity, and low canopy temperatures under heat-stress conditions. Meanwhile, one family developed from accession collected from southern lineage demonstrated high biomass, harvest index, and seed number under heat-stress conditions. These three families produced heat-tolerant lines containing unique genomic segments from their wild emmer wheat parents that were absent from their heat-sensitive sister lines. The wild emmer-derived families evaluated in this study showed significant intraspecific variation for heat stress tolerance and demonstrated their potential to enhance wheat adaptation to heat stress.

## Materials and methods

### Plant materials

We used an intraspecifically diverse set of wild emmer wheat (*T. turgidum* ssp. *dicoccoides*) accessions to develop nine WED families. These WED families were developed by crossing and backcrossing nine wild emmer accessions provided by the National BioResource Project–Wheat, at Kyoto University with durum wheat (*T. turgidum* ssp. *durum*) cultivar ‘Miki 3’, an elite cultivar developed by ICARDA (Pedigree: Stj3//Bcr/Lks4, released as a variety in Syria and Lebanon with different names). The nine wild emmer accessions were KU-108-1, KU-108-4, KU-108-5, KU-14474, and KU-14532 from the southern lineage, and KU-8808, KU-8810, KU-8814, and KU-8815 from northern lineage of the natural distribution (Ozkan et al., 2005; Özkan et al., 2011; Peng et al., 2011). The detailed procedures of WED families development were provided by Balla et al. (2022a). Briefly, the nine wild emmer accessions were crossed and backcrossed to ‘Miki 3’ to produce 9 BC_1_F_1_ families. From each family, 10 plants were selected and 10 self-pollinated seeds from each plant were mixed and sown to give a population of 900 BC_1_F_2_ plants. The population was then advanced to BC_1_F_3_ where 501 plants with good agronomic performance were selected in the field of the Arid Land Research Center, Tottori University, Japan. This population was named as Multiple Derivative Lines (MDLs) (Balla et al., 2022a). The 501 BC_1_F_4_ plants of the MDL population were then subjected to preliminary evaluation under heat stress conditions in Wad Medani, Sudan. Based on phenology and grain yield under the heat stress condition at Wad Medani, we selected 178 BC_1_F_6_ lines from the MDL population. The 178 BC_1_F_6_ lines were genotyped, and their pedigree was identified (Balla et al., 2022a). According to the pedigree analysis, the 178 BC_1_F_6_ lines were separated into nine families corresponding to the wild emmer wheat accessions. These families were named wild emmer derivative (WED) families. The numbers of lines within the nine WED families were not equal because of successive selection during the development of these lines, especially under the heat stress condition where all lines with high vernalization requirements were dropped. The list of the families with their lines and parents is shown in **Supplementary Table S1**. The recurrent parent ‘Miki 3’ served as a control for each family.

### Phenotyping environments

As a single composite, the nine WED families were evaluated under four field environments: one in Japan and three in Sudan. In Japan, the experiment was conducted in Tottori at the field of the Arid Land Research Center, Tottori University (35°32′N, 134°13′E, 11 m a.s.l.), hereafter abbreviated as TOT. In Sudan, the nine WED families were tested under contrasting field conditions ranging from the relatively cool environment at Dongola in Northern Sudan (19°08′N, 30°27′E, 239 m a.s.l; abbreviated as DON) to the continuous heat stress environment in the central clay plain, Gezira State, Central Sudan. At the Gezira State, we used the experimental field of the Gezira Research Station Farm (GRSF), Agricultural Research Corporation, Wad Medani (14°24′N, 29°33′E, 407 m a.s.l). At GRSF, two sowing dates were used: a normal (optimum) sowing date (NSD, sown on November 27, 2019) and a late sowing date (LSD, sown on December 23, 2019).

The testing location in TOT was considered an optimum wheat-growing environment, while DON was considered a non-stress wheat environment in Sudan. On the other hand, GRSF in Sudan is characterized as a dry and hot irrigated environment with maximum temperatures consistently above 30°C. The normal sowing date (NSD) was considered a moderate heat stress environment, whereas the late sowing date (LSD) was considered a severe heat stress environment.

All field descriptions, management, fertilization, irrigation, and experiment plot sizes at TOT were the same as described by Elhadi et al. (2021b) and Balla et al. (2022b). At all locations in Sudan, field description, management, fertilization, irrigation, and experimental plot size were the same as described by Mohamed et al. (2022) and Balla et al. (2022b). All field experiments were sown in alpha-lattice designs with two replications.

### Data collection

The data were collected for phenology, morphology, leaf physiology, biomass, grain yield, and some yield-related traits. Days to heading (DH) was recorded as the days from sowing to when 50% of spikes merged from the leaf sheath. Days to maturity (DM) was recorded when 90% of spikes in a plot showed total loss of green color, whereas the difference in days between DM and DH (DM-DH) was considered as grain filling duration (GFD). Plant height (PHT) was measured as the distance from the ground level to the top of the spike, excluding awns. Leaf physiological traits included canopy temperature at heading (CTH), chlorophyll contents at heading (CHLH), chlorophyll content at physiological maturity (CHLM), and chlorophyll degradation (CHLD) as the ratio of CHLM to CHLH 100*(CHLH-CHLM)/CHLH only under NSD and LSD. The canopy temperature was measured using a portable infrared thermometer in the afternoon (12:00-15:00) of sunny days. Chlorophyll content was measured from three random flag leaves using a portable chlorophyll meter (SPAD-502, Konica-Minolta, Japan). The biomass (BIO, kg ha^-1^) and the grain yield (GY, kg ha^-1^) were measured as the total above-ground weight and grain weight from the plot and then converted to kg ha^-1^. Harvest index (HI, %) was measured as the ratio between BIO and GY 100*(GY/BIO). Thousand kernel weight (TKW, g) and seed number per spike (SN) were measured using a sample of 10 spikes selected randomly from the two central rows in each plot.

### Stress tolerance index

To identify heat-tolerant families, the STI was calculated based on GY using the equation below (Fernandez, 1992).


$$\text{STI}-\text{GY}=\frac{\left({\text{Y}}_{\text{N}})({\text{Y}}_{\text{S}}\right)}{{\left({\text{Y}}_{\text{M}}\right)}^{2}}$$

where ${\text{Y}}_{\text{N}}$ is the GY of each line in each family under non-stress conditions; ${\text{Y}}_{\text{S}}$ is the GY of each line in each family under heat stress conditions, and ${\text{Y}}_{\text{M}}$ is the mean GY under non-stress conditions.

### Statistical analysis

We performed the analysis of variance (ANOVA) of all traits for all lines within the families in each location separately. For combined analysis, restricted maximum likelihood (REML) variance components analysis was conducted for all studied traits using GenStat 23^rd^ edition (http://www.genstat.co.uk). The REML analysis used the WED families, environments, and their interaction as fixed effects. The environments, replications, and sub-blocks were used as random effects. The grain yield of each WED families across environments was compared using Tukey’s honestly significant difference (HSD) test. The GY-trait correlation coefficient was calculated in each family using IBM SPSS Statistic for Windows, V. 29 (IPM Corp., Armonk, NY, USA). The variable principal component analysis (PCA) and the biplot PCA for all lines within the families across environments were performed using FactoMineR and Factoextra R packages (Lê et al., 2008). The heatmap plots for lines of three selected families (families 6, 8, and 9) were constructed using the Pheatmap package in R software (Barter and Yu, 2018). The Additive Main Effect and Multiplicative Interaction (AMMI) model was used for the GY data from four environments using GenStat 23^rd^ edition (http://www.gensts.co.uk). The AMMI2 biplot based on the first two interaction principal component analysis (IPCAs) scores for all lines within the families across environments, was generated using the metan R package (Olivoto and Lúcio, 2020). The AMMI model combines ANOVA with multivariate PCA, using standardized residuals from the ANOVA to analyze trait data by isolating experimental error and genotype × environment interaction effects, excluding the main effects of genotype and environment (Demelash, 2024).

The AMMI model equation is:


Yіj=μ+αj+βj+∑m=1m'λmϒmjδmj+θij+ϵj

where: Yij = the mean yield of line і in environment j , μ  = the grand mean of the yield, ${\text{α}}_{\text{j}}$ = the deviation of the line mean from the grand mean, ${\text{β}}_{\text{j}}$ = the deviation of the environment mean from the grand mean, m′ = the number of PCA axis retained in the model, λ_m_ = the singular value for the PCA, ${\text{ϒ}}_{m}$ = is the i-th line PCA score for the axis m, ${\text{δ}}_{\text{mj}}$ = is the j-th environment PCA sore for the axis m, ${\text{θ}}_{\text{ij}}\;$ = the AMMI residual, and Ƹ_j_ = the residuals.

From the AMMI estimate for grain yield in each environment, the percent contribution of each family in the top-yielding 25 lines was calculated. Likewise, the contribution of each family in the top stable 25 lines based on AMMI stability value (ASV) across all environments was also calculated.

### DArT-sequence genotyping and molecular analysis

Fresh leaf tissue (~50 mg) from 6-day-old seedlings from each genotype was used for DNA extraction via modified CTAB method (Saghai-Maroof et al., 1984). The DNA samples were sent to the Diversity Array Technology Pty Ltd., Australia (http://www.diversityarrays.com) for a genome scan using the DArT-seq platform. Restriction fragments from each genotype were sequenced and aligned to durum wheat reference genome ‘Svevo’ RefSeq v.1.0 to generate Silico DArT or SNP markers (Maccaferri et al., 2019). We used Silico DArT markers to perform graphical genotyping for selected lines within three selected families 6, 8, and 9. The dominant DArT markers were scored as presence (‘1’) homozygous SNP allele or absence (‘0’) homozygous reference allele. Only the mapped and polymorphic markers between the recurrent parent ‘Miki 3’ and the wild emmer parents were used for the graphical genotyping. Markers were ordered according to their position in each chromosome, and a conditional formatting function in Microsoft Excel was used to plot polymorphic markers between ‘Miki 3’ and wild emmer parents. A total of 7,275 SNP markers with no missing genotype (100% call rate) were used for principal component analysis for three selected families 6, 8, and 9. SNP markers, which are codominant, were scored as (‘0’) homozygous reference allele, (‘1’) homozygous SNP allele, or (‘2’) heterozygous. Marker-trait associations previously identified under NSD and LSD were used to determine allele contributions from wild emmer wheat or ‘Miki 3’ for three selected families 6, 8, and 9. Detailed methods for the GWA study were described by Balla et al. (2022b).

## Results

### Temperature data of four environments

**Figure 1:**
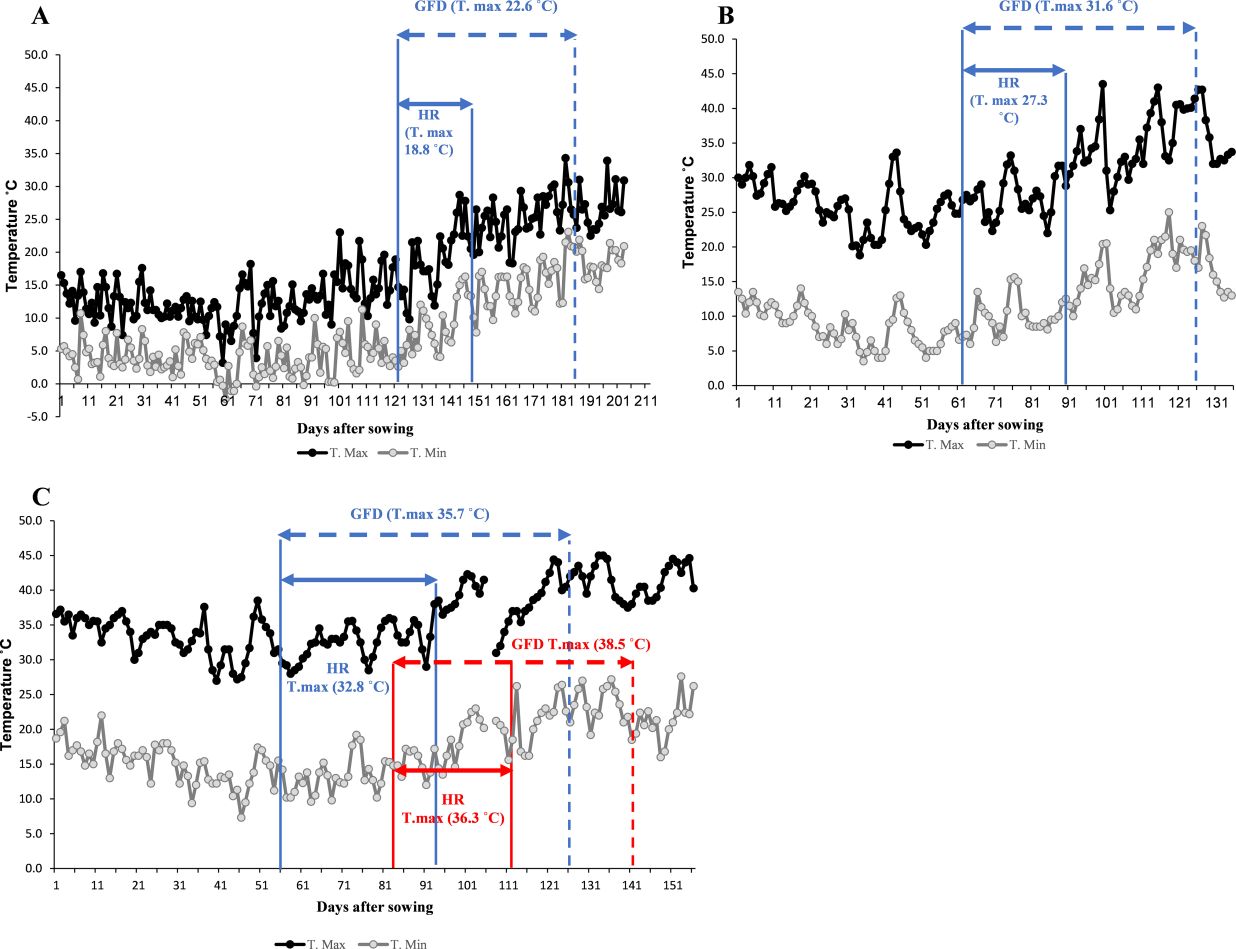
Daily maximum (T. Max) and minimum (T. Min) air temperature of the four environments used to evaluate the wild emmer derivative families. **(A)** Tottori (TOT), **(B)**, Dongola (DON), **(C)**, Wad Medani normal sowing date (NSD), and Wad Medani late sowing date (LSD). The horizontal sold blue arrows indicate the average maximum air temperatures (T. max) during heading range (HR) and dashed blue arrows during grain filling duration (GFD), respectively. HR in LSD indicated by sold red arrow whereases GFD indicated by dashed red arrow in panel **(C)**.

The testing locations showed a gradient increase in temperatures during the heading and grain-filling stages, progressing from optimum conditions at TOT and DON to the more stressful NSD and LSD environments at Wad Medani. The average maximum air temperatures during the heading and grain filling stages were 18.8°C, 22.6°C in TOT, and 27.3°C, 31.6°C in DON, respectively (**Figures 1A, B**). Under NSD, the average maximum temperatures during heading rose to 32.8°C and 35.7°C during grain filling. Under LSD, the average maximum temperatures were further increased to 36.3°C during the heading time and 38.5°C for grain filling duration (**Figure 1C**).

### Effect of heat stress on the performance of WED families

To assess genetic diversity within each family, the nine WED families were phenotypically characterized by different phenological and morpho-physiological traits, grain yield, and some related traits.

The means and ranges of the WED families and the recurrent parent ‘Miki 3’ in each environment for all traits studied are shown in **Supplementary Table S2**. Heat stress caused significant decreases in most of the traits studied in the nine WED families (**Supplementary Table S2**).

The REML analysis revealed that the effects of the environment on all traits studied were highly significant (**Supplementary Table S3**). The effect of environment on CHLD calculated for the two-heat stress environment was significant at *P<*0.05. Significant differences were found among the nine families in GY, DH, DM, GFD, PH, CHLH, and SN (**Supplementary Table S3**). However, no significant differences were detected between the families in BIO, HI, TKW, CHLM, and CHLD. The interactions between families by environments (F × E) showed significant differences for all studied traits except DH, DM, PHT, and CHLD (**Supplementary Table S3**).

**Figure 2:**
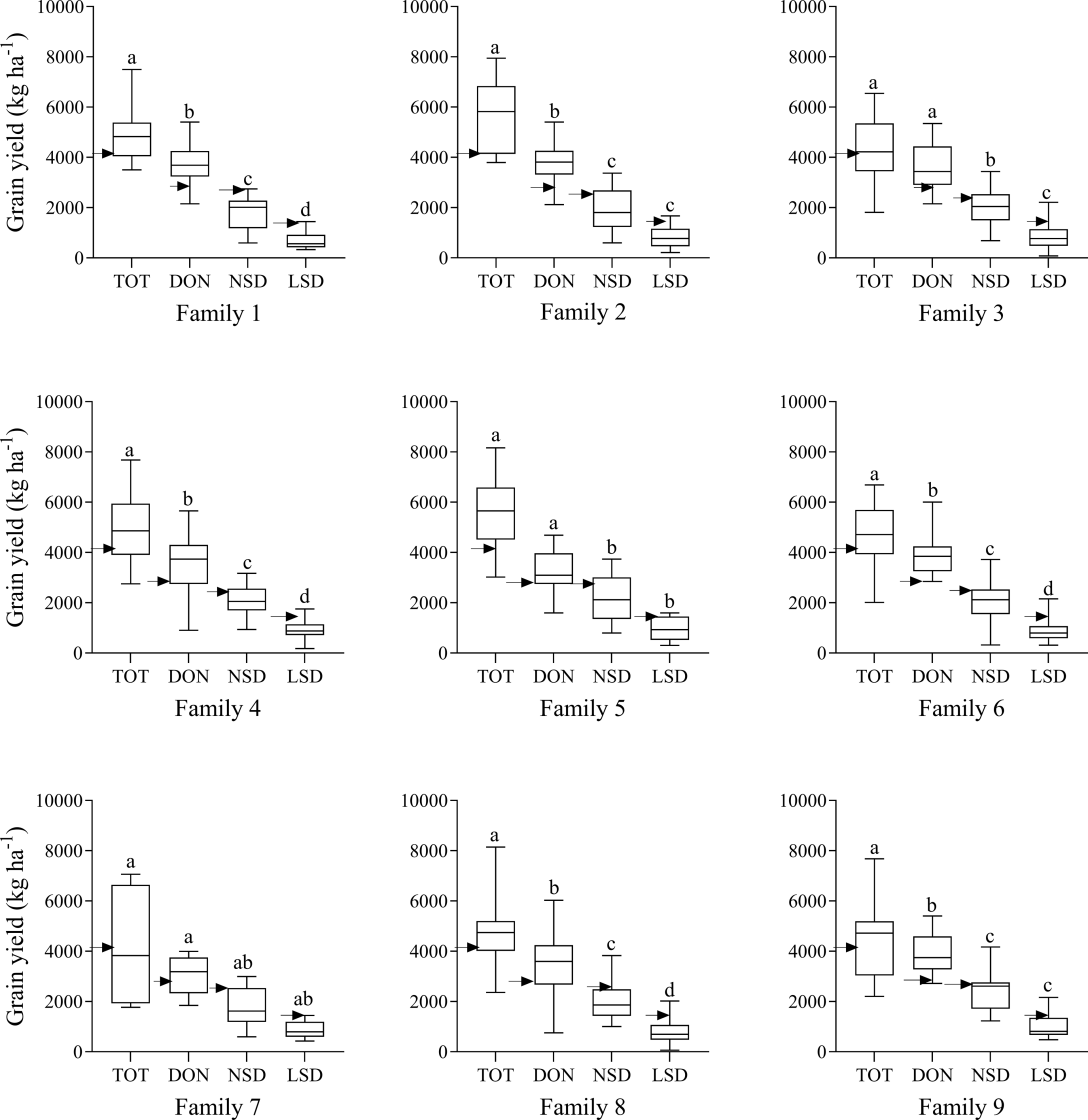
Grain yield of the wild emmer derivative families evaluated at Tottori (TOT), Dongola (DON), Wad Medani normal sowing date (NSD), and Wad Medani late sowing date (LSD). Black arrows indicate the position of the recurrent parent ‘Miki 3’. Boxes show medians and interquartile range, and whiskers show range. Environments were compared using Tukey’s honestly significant difference test at *P*< 0.01. The same letters indicate no significant differences between environments, whereas different letters indicate significant differences between environments.

Grain yields at DON were significantly reduced in most families compared to those at TOT (**Figure 2**). Likewise, compared to DON, significant reductions were found in grain yield at NSD in Wad Medani for all families except family 7. High temperatures during the grain filling in LSD at Wad Medani significantly (*P*< 0.001) reduced the GY for most of the WED families (five out of the nine WED families) compared with that of normal sowing (NSD) (**Figure 2**).

There was high variation in the performance of the WED families for grain yield across environments (**Table 1**). At TOT and DON, the highest mean GY values were recorded by family 2 (5822 and 3967 kg ha^-1^, respectively). On the other hand, the lowest GY values in both environments were recorded for family 7 (**Table 1**). Under the more stressful NSD and LSD, the highest mean GY values were obtained by family 9 (2192 and 978 kg ha^-1^, respectively). In contrast, the lowest GY values were obtained by family 7 and family 1 at NSD and LSD, respectively (**Table 1**). Notably, under NSD and LSD, some lines in families 6 and 8 demonstrated significantly higher GY than the recurrent parent ‘Miki 3’ and even surpassed lines from family 9 under LSD. The relative performance for GY of each family moving from optimum conditions at TOT to DON and from DON to stress conditions at NSD or from NSD to LSD was calculated and shown in **Table 1**. The highest GY reduction from optimum conditions at TOT or DON to more stressful conditions at NSD or LSD was obtained by family 2. Conversely, the lowest GY reduction from optimum condition at DON to heat stress environments at NSD and LSD was exhibited by family 9 (**Table 1**).

**Table 1:** Mean of grain yield (GY) and relative performance (RP) of nine wild emmer derivative families evaluated under Tottori (TOT), Dongola (DON), Wad Medani normal sowing date (NSD), and Wad Medani late sowing date (LSD) during season 2019/2020.

Family	GY, TOT	GY, DON	GY, NSD	GY, LSD	RP (TOT to DON)	RP (TOT to NSD)	RP (TOT to LSD)	RP (DON to NSD)	RP (DON to LSD)	RP (NSD to LSD)
Family 1	4947	3768	1663	649	76.17	33.62	13.12	44.13	17.22	39.03
Family 2	5822	3967	1949	810	68.14	33.48	13.91	49.13	20.42	41.56
Family 3	4266	3765	2037	865	88.26	47.75	20.28	54.10	22.97	42.46
Family 4	5139	3763	2057	917	73.22	40.03	17.84	54.66	24.37	44.58
Family 5	5566	3206	2185	908	57.60	39.26	16.31	68.15	28.32	41.56
Family 6	4686	3843	2011	868	82.01	42.92	18.52	52.33	22.59	43.16
Family 7	4016	3056	1609	860	76.10	40.06	21.41	52.65	28.14	53.45
Family 8	4845	3560	2063	828	73.48	42.58	17.09	57.95	23.26	40.14
Family 9	4701	3872	2192	978	82.37	46.63	20.80	56.61	25.26	44.62

### AMMI model analysis

To assess the contribution of each family in various environments and evaluate their stability, we conducted an AMMI analysis using GY data from four environments.

The AMMI (ANOVA) revealed that GY was significantly (*P<* 0.001) affected by genotype (G), environment (E), and G × E interaction (GEI), which explained 9.06, 71.86, and 19.08% of the variation due to the treatment, respectively (**Table 2**). The first two principal components (PCs) were highly significant and accounted for 90.2% of the variation due to GEI (**Table 2**; **Supplementary Figure S1**).

**Table 2:** The analysis of the variance of grain yield of the wild emmer dervative families tested across four environments in the 2019/2020 season using Additive Mean Effect and Multiple Interaction (AMMI) model.

Source	d.f.	s.s.	m.s.	% explained
Total	1439	5359924536	3724756	
Treatments	719	4743535352	6597407	
Genotypes	179	429585724	2399920^***^	9.06
Environments	3	3408887759	1136295920^***^	71.86
Block	4	50259950	12564987	
Interactions	536	905061869	1688548^***^	19.08
IPCA 1	181	596305691	3294507^***^	65.89
IPCA 2	179	219612861	1226887^***^	24.26
Residuals	176	89143316	506496^ns^	
Error	706	566129234	801883	

d.f, Degree of freedom; s.s, Sum of the square; m.s, Mean sum of squares, ‘***’,

significant at p-value< 0.001; ns, not significant at *p*-value; IPCA, interaction principal component analysis.

The AMMI2 biplot is divided into four quadrants, with the families located near the ordinate axis indicating general adaptation to the environments. The optimum environments TOT and DON exhibited stronger interactions. However, the more stressful environments NSD and LSD showed moderate to the least GEI, respectively (**Supplementary Figure S1**).

Based on the AMMI estimate, families 4, 6, and 8 demonstrated resilience to heat stress, maintaining performance across different environments. For instance, under optimum conditions at TOT, 28% and 20% of the top 25 performing lines were from families 4 and 8, respectively. At DON, 36% of the top-ranking 25 lines were from family 6 (**Supplementary Table S4**). Under moderate heat stress (NSD), 24 and 20% of the top 25 lines were from families 6 and 4, respectively. Under severe heat stress (LSD), both family 4 and family 6 contributed by 28 and 24%, respectively, in the top-ranking 25 lines (**Supplementary Table S4**). Across the four environments, families 8 and 6 contributed to the top-ranking lines by 24 and 16%, respectively (**Supplementary Table S4**).

When considering the top-ranking 25 lines based on AMMI stability value (ASV) across all environments, the maximum contribution was from both families 6 and 8 (24%), followed by family 4 (16%; **Supplementary Table S4**). These families also exhibited a lower degree of GEI, as their lines were generally positioned close to the origin in the AMM2 biplot. However, some lines within these families have higher GEI, as they were located farther from the origin (**Supplementary Figure S1**).

### Screening for heat stress tolerance among WED families

To identify heat tolerant families, the heat stress tolerance index (STI) was calculated twice; the first STI (STI1-GY) was calculated considering Dongola (DON) as a non-stress environment and normal sowing date (NSD) as a stress environments. The second STI (STI2-GY) was calculated considering NSD and LSD at Wad Medani as non-stress and stress environments, respectively.

**Figure 3:**
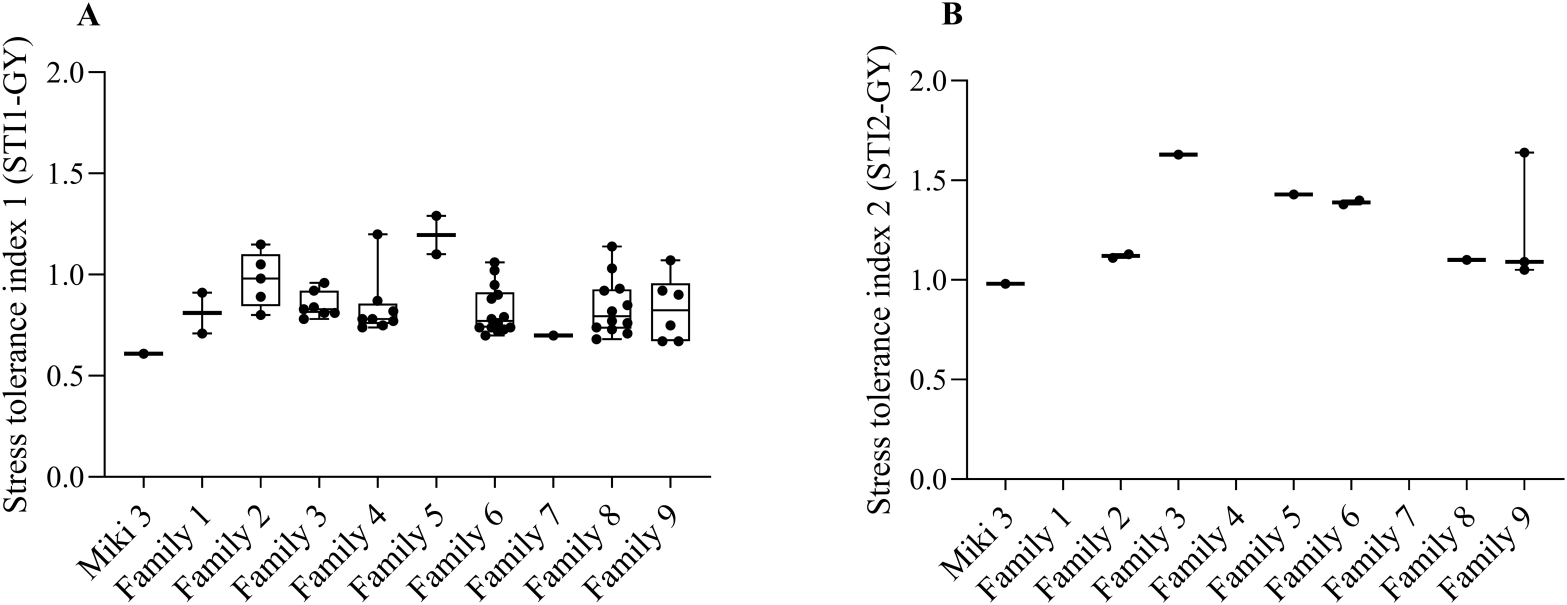
Stress tolerance index (STI) calculated based on grain yield (GY) for the wild emmer derivative (WED) families and the backcross parent ‘Miki 3’. **(A)** STI1-GY, stress tolerance index calculated based on grain yield from Dongola versus that of Wad Medani normal sowing date. **(B)** STI2-GY, stress tolerance index calculated based on grain yield from Wad Medani normal sowing date versus that of Wad Medani late sowing date. The WED families and the backcross parent ‘Miki 3’ were shown on the x-axis of each plot. Boxes show medians and interquartile range, and whiskers show range.

The STI1-GY results showed that 57 lines had higher STI values than the recurrent parents and were distributed among all families. However, families 3, 4, 6, 8, and 9 contributed with 7, 8, 14, 12, and 7 lines, respectively, which represented 31.8, 29.6, 37.8, 36.3, and 46.1% of the total lines in each family (**Figure 3A**). For STI2-GY, only 10 lines demonstrated higher STI than ‘Miki 3’, distributed among six families (**Figure 3B**). Families 2, 6, and 9 contributed with 2, 2, and 3 lines, respectively (**Figure 3B**). Combining both STIs, the highest number of heat tolerant lines stemmed from family 6 (16), followed by family 8 (13), and family 9 (9), which represent 21.6, 19.6, and 34.6%, respectively. Therefore, we considered families 6, 8, and 9 as heat stress tolerant families because they consistently showed lines with high STI values at both STI1-GY and STI2-GY.

### Association between GY and other traits in WED families

At the level of each WED family, the correlation of GY with the studied traits in the four environments is shown in **Supplementary Tables S5–S8**. The GY and BIO were correlated in most of the WED families under all environments except at DON, where it is only observed in families 4 and 8 (**Supplementary Table S6**). The correlation between GY and HI was found in the majority of the WED families at TOT, NSD, and LSD. At NSD and LSD, a positive correlation between GY and STI was evident for all WED families, except family 7 under LSD (**Supplementary Table S7, S8**).

The correlation between GY and other traits was found to be family-specific. For instance, at NSD, GY correlated with SN in families 4 and 6, TKW and GFD in family 6, and CHLM in family 1 (**Supplementary Table S7**). Conversely, negative correlations were found between GY and CTH in families 6 and 8 at NSD and LSD (**Supplementary Tables S7, S8**). At LSD, GY was correlated with TKW in families 4 and 8 (**Supplementary Table S8**).

### Principal component analysis

**Figure 4:**
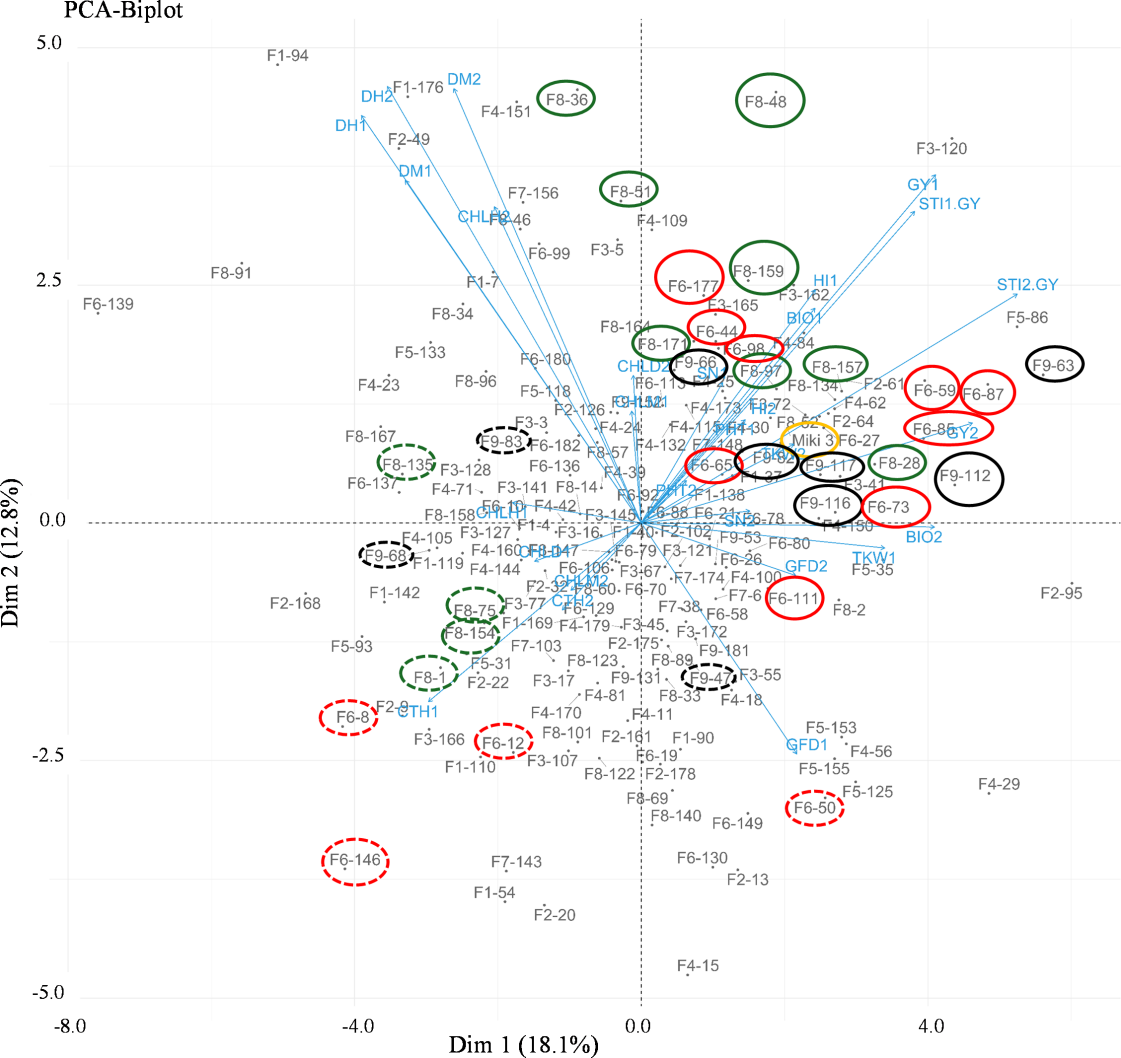
Biplot of the principal component analysis (PCA) showing different trends in the wild emmer derivative (WED) families and their evaluated traits at Wad Medani normal sowing date (NSD) and Wad Medani late sowing date (LSD). The numbers 1 and 2 attached to the trait in the PCA indicate the trait evaluated at NSD and LSD, respectively. High tolerant lines are circled by solid red, black, and green colors whereas sensitive sister lines are circled by dashed red, black and green colors. Miki 3 the recurrent parent circled by yellow color on the PCA. F1 to F9 attached to the WED lines corresponded to the nine families. STI1-GY, stress tolerance index calculated based on the grain yield from Dongola versus that of Wad Medani normal sowing date; STI2-GY, stress tolerance index calculated based on grain yield from Wad Medani normal sowing date versus that of Wad Medani late sowing date; DH, days to heading; DM, days to maturity; GFD, grain filling duration; CHLH, chlorophyll at heading; CHLM, chlorophyll at maturity; CHLD, chlorophyll degradation; GY, grain yield; BIO, biomass; TKW, thousand kernel weight; HI, harvest index; SN, seed number/spike; PHT, plant height.

To explore the clustering patterns of the lines within their respective families and identify any overlap, we constructed a biplot based on the principal component analysis using the data of the two heat stress environments (NSD and LSD at Wad Medani; **Supplementary Figure S2** and **Figure 4**). The PC1 and PC2 explained 18.1 and 12.1% of the variation, respectively (**Figure 4**). The same trait is found in both environments, which are mostly associated with each other. The WED families clustering showed overlap among the families; however, some lines did not tightly clustered within their families. Several lines belonging to some families closely associated with the GY and STI-GY were identified and separated from the recurrent parent. For instance, families 6, 8, and 9 contributed with more heat tolerant lines under both heat stress environments, although some lines belonging to these families were also found to be sensitive to heat stress with high CTH. On the other hand, most lines from other families showed low STI-GY and high CTH, indicating susceptibility to heat stress.

**Figure 5:**
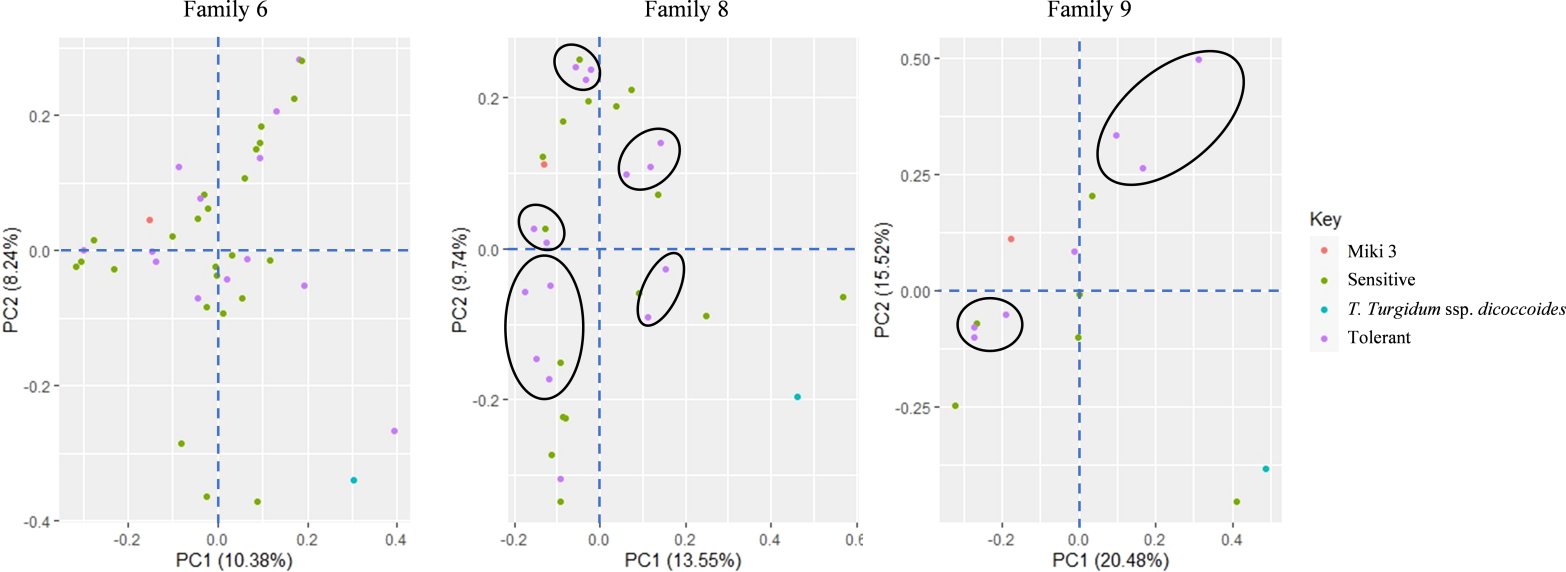
Principal component analysis (PCA) of diversity in three wild emmer derivative families 6, 8, and 9 based on 7 275 SNP markers. The close tolerant lines are circled by black color.

At the genotypic level, we performed PCA on families 6, 8, and 9 using SNP markers to understand the contribution of the wild emmer allele to the tolerant lines in these families (**Figure 5**). The first and the second components accounted for 18.6, 23.3, and 36.0% of the variation in families 6, 8, and 9, respectively. In family 6, the consistent tolerant lines from both STI1-GY and STI2-GY showed substantial overlaps with sensitive lines on the PCA plot (**Figure 5**). Conversely, in families 8 and 9, the tolerant lines formed distinct clusters from the sensitive lines on the PCA (**Figure 5**).

### Heat stress tolerance-associated trait in families 6, 8 and 9

**Figure 6:**
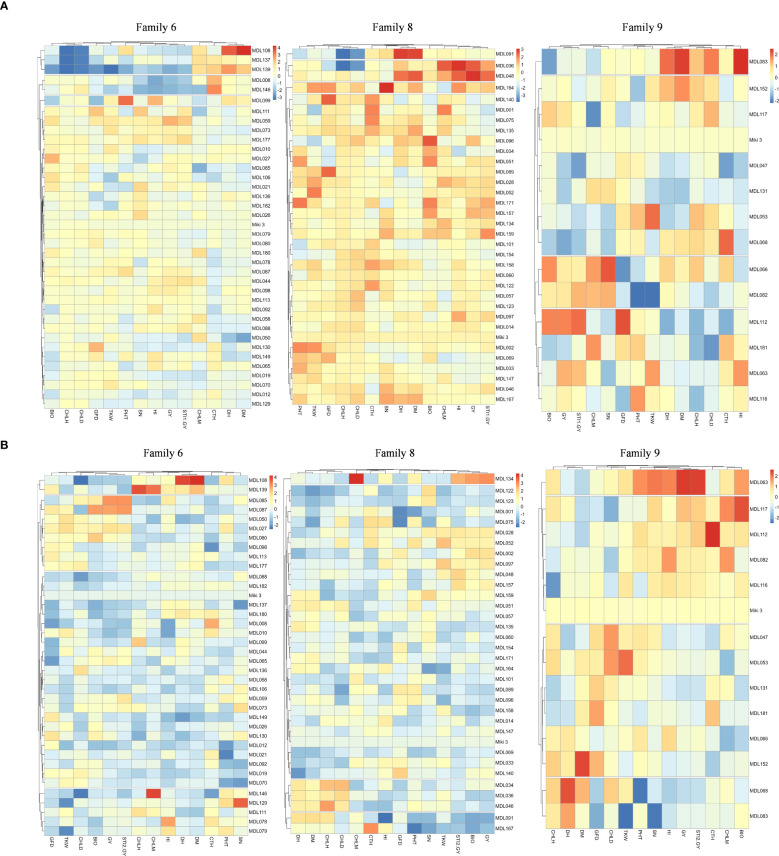
**(A)** Heatmap explained heat stress tolerance-associated trait in tolerant families 6, 8, and 9 evaluated under normal sowing date at Wad Medani, Sudan. The recurrent parent ‘Miki 3’ was plotted as blank in the heatmaps. STI1-GY, stress tolerance index calculated based on the grain yield from Dongola versus that of Wad Medani normal sowing date; DH, days to heading; DM, days to maturity; GFD, grain filling duration; CHLH, chlorophyll at heading; CHLD, chlorophyll degradation; CHLM, chlorophyll at maturity; BIO, biomass; TKW, thousand kernel weight; HI, harvest index; SN, seed number/spike; CTH, canopy temperature at heading stage. **(B)** Heatmap explained heat stress tolerance-associated trait in tolerant families 6, 8, and 9 evaluated under late sowing date at Wad Medani, Sudan. The recurrent parent ‘Miki 3’ was plotted as blank in the heatmaps. STI2-GY, stress tolerance index calculated based on grain yield from Wad Medani normal sowing date versus that of Wad Medani late sowing date; DH, days to heading; DM, days to maturity; GFD, grain filling duration; CHLH, chlorophyll at heading; CHLD, chlorophyll degradation; CHLM, chlorophyll at maturity; BIO, biomass; TKW, thousand kernel weight; HI, harvest index; SN, seed number/spike; CTH, canopy temperature at heading stage.

To identify heat stress-tolerant traits in families 6, 8, and 9, we constructed a heatmap for the evaluated traits in these families under NSD and LSD (**Figures 6A, B**).

Under NSD, each family was grouped into four main clusters (**Figure 6A**). In family 6, the STI1-GY was closely associated with GY, HI, and SN. In family 8, the STI1-GY was closely associated with GY, HI, and CHLM, while in family 9, the STI1-GY was linked with GY and BIO (**Figure 6A**).

Under LSD, the clustering pattern remained, but the association varied slightly (**Figure 6B**). In families 6 and 8, STI2-GY was closely associated with GY and BIO, with an additional association with TKW in family 8. In family 9, STI2-GY was associated with GY, HI, SN, and PHT (**Figure 6B**). Under both NSD and LSD, most lines in family 6 exhibited medium to low CTH, as did the lines in family 8 under LSD (**Figures 6A, B**).

### Graphical genotyping for families 6, 8 and 9

**Figure 7:**
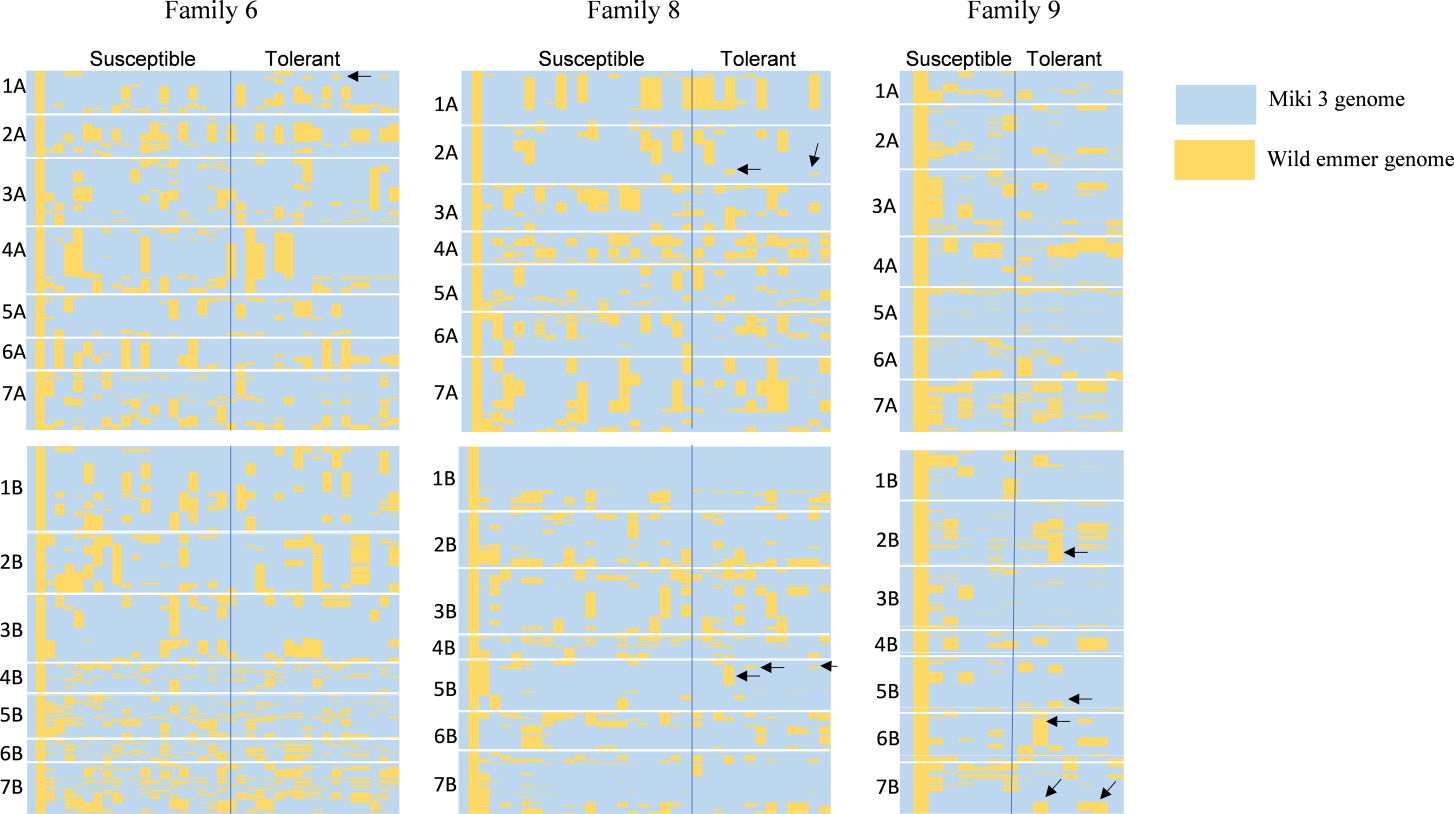
Graphical genotyping shows the genomic differences between tolerant and susceptible lines in three wild emmer derivative (WED) families 6, 8, and 9. In each family, the first column indicates the genome of the recurrent parent ‘Miki 3’ (pale blue), and the second column is the wild emmer genome (yellow color). Conditional formatting in Microsoft Excel generated the plots from polymorphic markers between ‘Miki 3’ and wild emmer. Blue and yellow colors spread in each family indicate the ‘Miki 3’ and wild emmer genomes, respectively. The letter on the left of each family indicates chromosomes. The black arrows in tolerant lines in each family showed genomic segments different from that of susceptible ones.

To pinpoint genomic regions associated with heat stress adaptation, we performed graphical genotyping between sensitive and tolerant lines in families 6, 8, and 9 (**Figure 7**). The analysis revealed distinct genomic differences between tolerant and susceptible lines across 14 chromosomes. For example, some tolerant lines in family 6 had introgressed segments on chromosome 1A from their wild emmer parent that were absent in their susceptible counterparts (**Figure 7**). In family 8, the tolerant lines possessed unique genomic segments on chromosomes 2A and 5B from wild emmer parent that were missing in susceptible lines (**Figure 7**). Similarly, the tolerant lines in family 9 featured different wild emmer segments on chromosomes 2B, 5B, 6B, and 7B, which were not present in the heat-sensitive sister lines at such chromosomes (**Figure 7**).

### Wild emmer allele contribution to the tolerant lines in families 6, 8, and 9

In our previous genome-wide association study of 178 BC_1_F_6_ of the WED families grown under NSD and LSD, we identified significant marker-trait associations (MTAs) that highlighted the role of wild emmer alleles in various tolerance traits (Balla et al., 2022b). This current study specifically focused on MTAs related to heat resilience, examining the role of wild emmer alleles in three selected tolerant families: families 6, 8, and 9 (**Supplementary Table S9**). The traits investigated were HI and CTH under both NSD and LSD, as well as BIO and CHLM under LSD. These traits were chosen due to their association with STI in these families (**Figures 4**, **6A, B**).

Under NSD, six MTAs originating from wild emmer genomes were identified for HI on chromosome 2B, with four linked to family 6 and two to family 8 (**Supplementary Table S9**). In addition, one MTA for HI on chromosome 7A was found to be specific to family 6. In LSD conditions, eight MTAs for HI were identified: four on chromosome 2B and two on chromosome 2A associated with both family 6 and family 8. An additional two MTAs for HI on chromosomes 7B and 6A were found to be specific to family 6 and family 8, respectively (**Supplementary Table S9**). For CTH under NSD, one MTA was identified on chromosome 7B, linked to both family 6 and 8, and another MTA on chromosome 6B was found to be specific to family 8. Under LSD conditions, three MTAs for CTH were identified: one on chromosome 3B linked to families 6 and 8, and two on chromosome 6A associated with family 9 (**Supplementary Table S9**).

For CHLM under LSD, one MTA from the wild emmer genome identified on chromosome 1A was specific to family 6. Regarding BIO under LSD, four MTAs originating from wild emmer genomes were identified on chromosomes 3A (three MTAs) and 6B (one MTA). These MTAs specifically linked to family 6 (**Supplementary Table S9**).

## Discussion

### WED families response to different levels of heat stress

Wheat improvement requires extensive exploration of potential genetic resources and a deep understanding of their adaptation to harsh environments, particularly high temperatures. Here, we elucidate the intraspecific variation of nine wild emmer derivative families in an elite durum wheat cultivar ‘Miki 3’ genetic background for heat stress tolerance. The nine wild emmer used to develop the families in this study originated from two lineages with a wide genetic diversity and were expected to harbor alleles/genes that could improve heat stress tolerance in wheat.

High genetic variation was detected among the WED families for most of the studied traits. The environmental effect on WED families performance was highly significant for all studied traits and F × E interaction for most traits (**Supplementary Table S3**). These results indicate that the nine WED families differentially responded to different environments and possessed high levels of phenotypic variation.

For most evaluated families, GY and other essential traits significantly reduced from TOT to DON, DON to NSD, or NSD to LSD (**Supplementary Table S2**) due to the gradient increase in temperature from optimum to heat stress environments (**Figure 1**). Under TOT and DON, family 2 achieved the highest GY, but this family showed a severe reduction in GY from optimum to heat stress conditions (**Table 1**). Conversely, family 9 showed the highest GY under NSD and LSD at Wad Medani and was one of the top three families for GY in DON (**Table 1**). Additionally, family 9 showed low GY reduction from optimum to heat stress conditions (**Table 1**). The high GY in family 2 under TOT was linked to high BIO, and under DON, high SN, HI, and TKW (**Supplementary Table S2**).

The high GY in family 9 under NSD and LSD was associated with its high SN, HI, and TKW compared with the recurrent parent ‘Miki 3’ and other families (**Supplementary Table S2**). As positive associations of GY with SN, HI, and TKW have been reported under heat stress conditions (Elbashir et al., 2017; Sukumaran et al., 2018; Elhadi et al., 2021a, 2021b; Itam et al., 2021; Balla et al., 2022), these traits would be a practical mechanism to maintain higher GY under heat stress. Families 2 and 9 were developed by wild emmer accessions from the southern lineage. The differences in the performance of the two families under optimum and heat stress conditions clearly showed the intraspecific variations among the accessions collected from the same lineage. Some wild emmer accessions collected from certain parts of the southern lineage were found to be adapted to hot and dry environments (Özkan et al., 2011; Nevo et al., 2012). Therefore, the wild emmer accession used to develop family 9 collected from such an area is expected to adapt well to heat stress conditions.

Based on AMMI estimates, the highest contributions to the GY were from families 4 (28%) and 8 (20%) at optimal conditions in TOT, while family 6 exhibited the best contribution (36%) to the GY under DON conditions. This suggests that these families performed well in favorable environments. Under both NSD and LSD stress conditions, families 4 and 6 revealed 28 and 24%, respectively, to the GY contribution (**Supplementary Table S4**). Importantly, neither family 4 nor family 6 was affected by high temperatures, maintaining stable performance in optimal conditions at TOT and DON, as well as in stressful environments at NSD and LSD.

According to AMMI estimates, lines with ASV scores close to zero exhibit low interaction effects and high stability across environments (Bishwas et al., 2021; Gupta et al., 2022). The most stable families identified were families 4, 6, and 8 (**Supplementary Table S6**), indicating that the accessions used to generate these families are less affected by environmental variability. This suggests that these families are valuable for breeding programs aimed at achieving grain yield stability under various levels of heat stress. However, some lines from such families have higher GEI (far from the origin), making them more sensitive to environmental changes and hence better suited to their favorable environment (**Supplementary Figure S1**). These findings also highlight the presence of intraspecific variation among wild emmer accessions, with some from the same lineage demonstrating different levels of stability and adaptability to different environmental conditions.

### Wild emmer contribution to heat stress tolerance

To identify the good source for heat stress tolerance, we calculated the stress tolerance index for all families using two levels of heat intensity (**Figures 3A, B**). STI1-GY and STI2-GY revealed that many tolerant lines originated from families 6, 8, and 9, suggesting these families are promising for developing new heat-tolerant cultivars. The wild emmer accessions used to generate families 8 and 9 originated from the southern lineage, while family 6 was derived from the northern lineage accession in Iraq (Balla et al., 2022a). Nevo et al. (2012) noting that the wild emmer accessions from the southern lineage in the Near East Fertile Crescent are rich in genetic resources for abiotic stress tolerance, including high temperatures. However, our study demonstrated significant intraspecific variation for heat stress tolerance for accessions from the southern lineage. Interestingly, while we examined four WED families derived from accessions collected from the northern lineage in Iraq, only one family (family 6) exhibited a high proportion of tolerance (21.6%), further confirming intraspecific genetic variation within the same region or country. Nevo et al. (1984) reported that the genetic diversity in the wild emmer is revealed between and within populations for economically significant traits. Family 6 may possess alleles adapted to high temperatures, as the eastern Mediterranean region, including Iraq, where wild emmer evolved, is characterized by long, hot, dry summers (Özkan et al., 2011; Huang et al., 2016; Mastrangelo et al., 2023). However, because the wild emmer is grown in very different climate conditions ranging from cold and humid to hot and dry in the Fertile Crescent region (Özkan et al., 2011; Mastrangelo et al., 2023), our families revealed a different genetic behavior in response to heat stress. Therefore, screening large accessions is crucial to identify valuable genes for breeding heat-tolerant varieties.

Interestingly, the tolerant lines identified in families 6, 8, and 9 were distinctly separated from their sensitive-sister progenies in the PCA biplot (**Figure 4**). This separation suggests the presence of remarkable heat resilience traits in these tolerant lines, which may be attributed to genes derived from wild emmer. For instance, the tolerant lines in family 6 under NSD and LSD tended to reduce CTH, a key indicator of heat stress response. Moreover, the high STI in family 6 may be associated with high HI and SN under NSD and high BIO under LSD (**Figures 4**, **6A, B**). Conversely, the high STI in family 8 was likely associated with high HI and CHLM under NSD, coupled with low CTH and high BIO under LSD. These results underscore the multifaceted nature of heat resilience in these families. Notably, certain lines from family 8 under LSD had higher TKW than their recurrent parent, indicating an ability to fill all grains within the spike under heat stress. On the other hand, the high STI in family 9 may be associated with high BIO under NSD and high HI, SN, and PHT under LSD. Thus, the heat resilient traits in these families vary, with some showing low CTH, high HI, BIO, SN, and some possessing stay green characteristics. Some identified traits such as low canopy temperature, high TKW, and the stay-green reported to be associated with higher tolerance to heat-irrigated environments in wheat (Pinto et al., 2010; Chandrasekhar et al., 2017; Kamal et al., 2019; Molero et al., 2023). However, to the best of our knowledge, this is the first report linking HI to heat stress tolerance in durum wheat. The trend toward higher HI may be associated with a tendency toward slightly shorter lines in family 6 and family 8. Because these lines have 75% from the recurrent parent, ‘Miki 3’ we believe they must contain one of the significant *Rht*, height-reducing genes. In addition, some of the tolerant lines with high HI in family 8 have relatively lower BIO under NSD (**Figure 6A**; **Supplementary Table S2**), possibly due to an increased grain assimilation rate, leading to higher HI (Reynolds et al., 2017). In contrast, susceptibility to heat stress was evident in certain lines from families 6, 8, and 9, characterized by elevated CTH, alongside diminished HI, SN, BIO, and TKW (dashed circled lines in **Figure 4**). This result indicates that the variability was between accessions from the same lineage or region.

Additionally, the genetic analysis revealed that the tolerant lines from families 8 and 9 clustered together (**Figure 5**), suggesting a common genetic basis for heat resilience, likely attributed to the shared allele contributions from the wild emmer. In contrast, the tolerant lines in family 6 showed substantial overlap with the sensitive lines (**Figure 5**), which may be attributed to the smaller introgression size from wild emmer in this family.

### Genomic regions from wild emmer associated with heat stress tolerance

Genotyping using polymorphic markers of the DArT-seq platform effectively distinguishes the heat-resilient lines from sensitive ones at various genomic regions (**Figure 7**). This result indicates the efficiency of utilizing wild emmer diversity in breeding for wheat heat stress tolerance (Peleg et al., 2005, 2009; Peng et al., 2013; Tsujimoto et al., 2015; Balla et al., 2022b). For example, heat-resilient lines in families 6, 8, and 9 possess genomic segments from their wild emmer parents on chromosomes 1A, 2A, 2B, 5B, 6B, and 7B, which were absent from heat-sensitive-sister lines at such chromosomes (**Figure 7**). This result highlights significant genomic variation within the recombinant lines of the same accession. Chromosome 1A has been linked to traits such as days to maturity, tolerance index, and plant height under heat stress in durum wheat (Sukumaran et al., 2018; Hassouni et al., 2019), as well as stay-green, maturity, and canopy temperature in bread wheat under heat stress conditions (Acuña-Galindo et al., 2015; Itam et al., 2021). In this study, unique segments on chromosome 1A were identified for some tolerant lines in family 6 (**Figure 7**). Therefore, the introgressed segments on chromosome 1A may contribute to heat stress adaptation in family 6 through low CTH or high HI, SN, and BIO.

Furthermore, the heat-tolerant lines from families 8 and 9 showed remarkable genomic segments from their wild emmer parents on chromosomes 2A, 2B, and 5B. These chromosomes possess critical phenology genes, such as the photoperiod response gene *Ppd-1* on the group 2 chromosomes and the vernalization requirement gene *Vrn-1* on the group 5 chromosomes (Whittal et al., 2018). Photoperiod and vernalization are essential for wheat adaptation to heat stress (Ferrara et al., 1998). However, Balla et al. (2022b) identified MTAs on chromosome 2A that regulate HI under heat stress conditions. In this study, we found that these MTAs carry favorable alleles from the wild emmer, which were linked to some lines in families 6 and 8 (**Supplementary Table S9**). Moreover, HI is recognized as a trait associated with heat stress tolerance in these families. In addition, other studies have identified QTLs controlling HI on chromosome 2A under both heat (Hassouni et al., 2019) and drought (Abdollahi Sisi et al., 2022) stress conditions.

In this study, some tolerant lines in family 8 under NSD exhibited high CHLM (**Figure 6A**). Thus, the introgressed segments from wild emmer on chromosome 5B in family 8 (**Figure 7**) likely contribute to heat adaptation by enhancing CHLM, a trait associated with heat stress tolerance in this family. Interestingly, Balla et al. (2022b) reported that a robust QTL controlling CHLM was mapped on chromosome 5B, with a beneficial allele for this trait derived from wild emmer wheat. Similarly, other studies (Acuña-Galindo et al., 2015; Bhusal et al., 2018) mapped several QTLs controlling chlorophyll content in wheat under heat stress on chromosome 5B. Additionally, Acuña-Galindo et al. (2015) performed a meta-analysis of reported QTLs associated with heat stress adaptation in wheat and found that chromosome 5B contained QTLs controlled HI, BIO, TKW, GY, and SN. Traits, such as HI and BIO were found to be heat-resilient traits in family 8 (**Figures 6A, B**), indicating that the introgressed wild emmer segment on chromosome 5B likely plays a critical role in heat stress adaptation through its effects on these traits.

Similarly, introgression segments on chromosomes 6B and 7B in family 9 may play a role in heat-resilient function. Sukumaran et al. (2018) reported that chromosome 6B harbors the QTL associated with the stress susceptibility index calculated based on grain yield. Hassouni et al. (2019) also identified QTLs on chromosome 6B controlling grain yield, seed number per spike, harvest index, biomass, and two stress tolerance indices of durum wheat grown under heat stress. In the current study, the tolerant lines from family 9 were associated with high BIO under NSD and high HI and SN under LSD (**Figures 6A, B**). Therefore, introgressed segments from wild emmer parent in family 9 on chromosome 6B may be linked to heat adaptation through these traits. Additionally, the introgressed segments on chromosome 7B in family 9 may be associated with heat stress adaptation through the stress tolerance index, as several QTLs for this index have been previously identified on chromosome 7B for durum wheat (Sukumaran et al., 2018) and for bread wheat (Mohammadi et al., 2008; Paliwal et al., 2012) grown under heat stress conditions.

In previous research, we identified several quantitative trait loci controlling heat resilient traits, with favorable alleles inherited from the wild emmer (Balla et al., 2022b). This study pinpoints the intraspecific variation among wild emmer accessions and specific traits inherited from the wild emmer wheat that confer heat resilience and adaptation to certain families. Pyramiding these traits from different WED families could provide a good opportunity to develop new high-heat-tolerant cultivars through multiple mechanisms. Currently, we are developing new molecular markers for some of these heat-resilient traits to facilitate marker-assisted selection for wheat cultivation under heat-stress conditions.

## Conclusion

In this study, nine wild emmer-derivative families were evaluated under varying levels of heat stress to assess intraspecific variation and identify heat-resilient traits. Two families, derived from accessions originating from both northern and southern lineages, displayed a high harvest index, increased chlorophyll content, and lower canopy temperature under heat stress. Another family derived from an accession from the southern lineage demonstrated high biomass, harvest index, and seed number under heat-stress conditions. These three families produced heat-tolerant lines containing introgressed chromosome segments, potentially associated with heat stress resilience.

The genetic differences observed between families highlight significant intraspecific variation among wild emmer wheat accessions in terms of heat stress tolerance. However, no consistent pattern was found between the northern and southern lineages. These results suggest that a broader evaluation of accessions is necessary to identify valuable genes for breeding heat-tolerant wheat. Besides the interspecific variation, harnessing genetic diversity within the intraspecific pool of wild emmer would be crucial for discovering genes that can improve heat stress resilience in wheat breeding programs.

## Data Availability

The datasets presented in this study can be found in online repositories. The names of the repository/repositories and accession number(s) can be found in the article/**Supplementary Material**.
