# Supplementary figures and images for: Intraspecific variation for heat stress tolerance in wild emmer-derived durum wheat populations
Source: Front Plant Sci. 2025 Jan 23;16:1523562. doi: 10.3389/fpls.2025.1523562 (PMC11798995; doi:10.3389/fpls.2025.1523562)

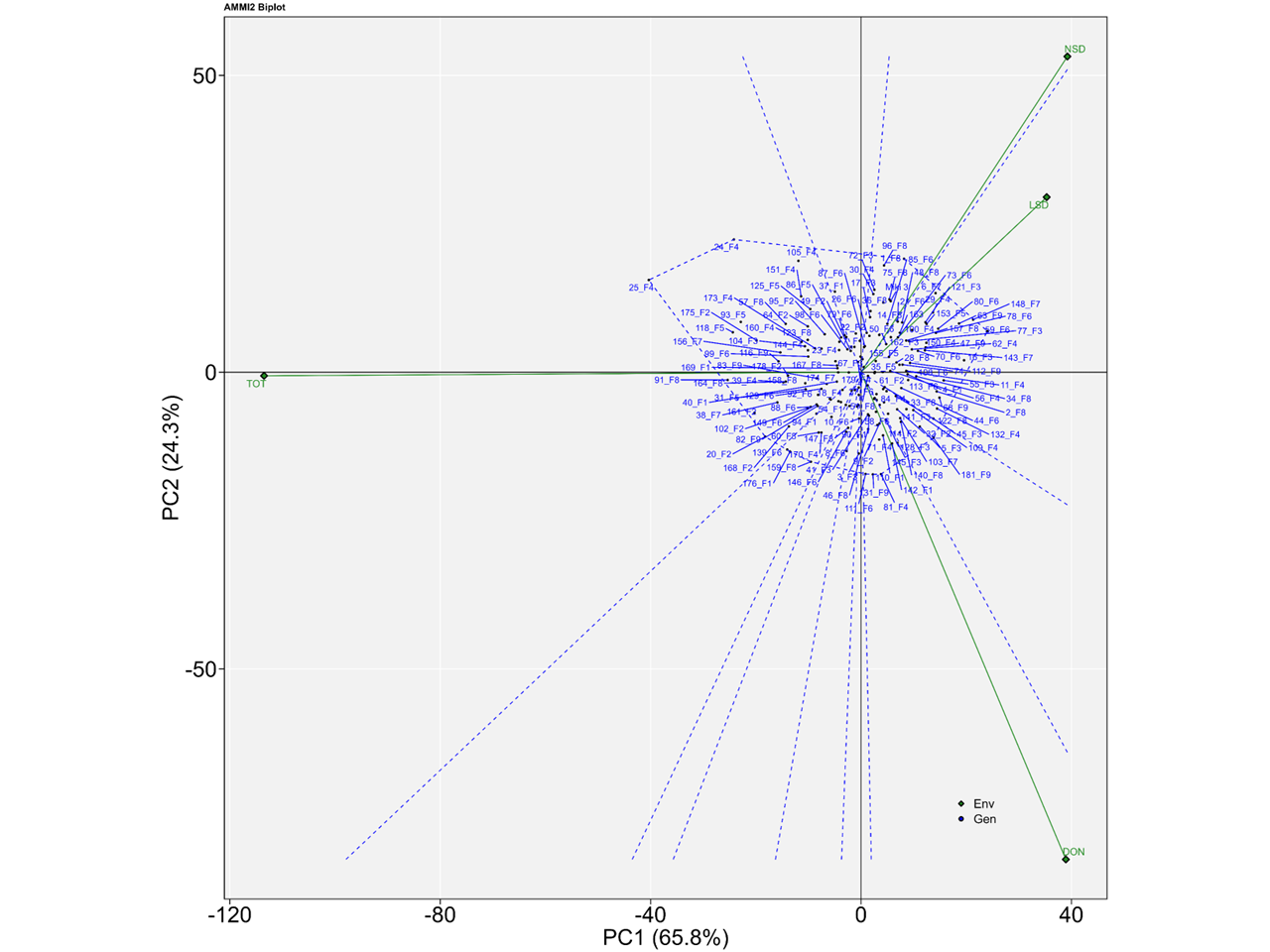

Supplement: Supplementary file 1 [file Image1.tif]

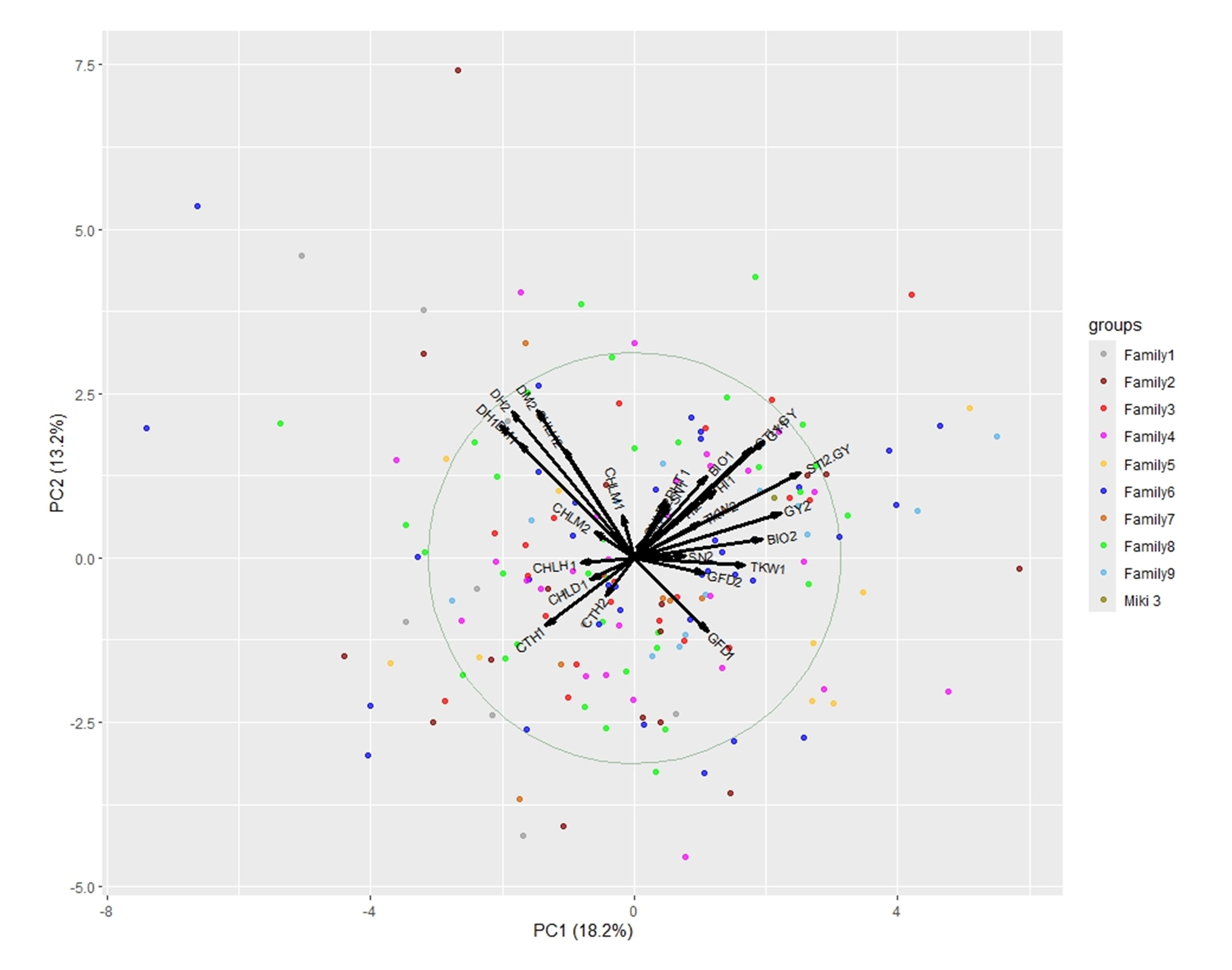

Supplement: Supplementary file 2 [file Image2.tif]
