# Supplementary material for: Intraspecific variation for heat stress tolerance in wild emmer-derived durum wheat populations
Source: Front Plant Sci. 2025 Jan 23;16:1523562. doi: 10.3389/fpls.2025.1523562 (PMC11798995; doi:10.3389/fpls.2025.1523562)
Supplement: Supplementary file 12 [file Table10.docx]

Supplementary Material

**
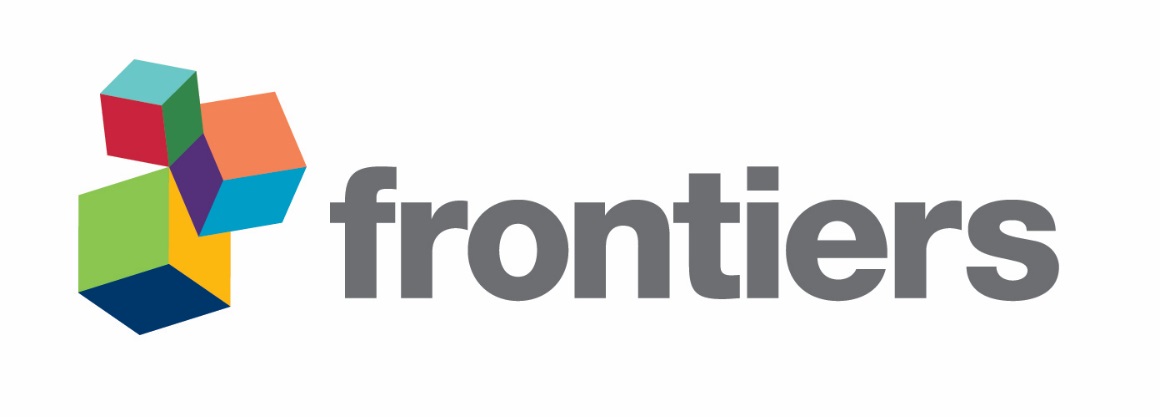
**

## Supplementary Figures

**Supplementary Figure 1.** AMMI2 biplot of the nine wild emmer derivative (WED) families and environments plotted against IPCA1 and IPCA2 using symmetrical scaling. F1 to F9 attached to the WED lines corresponded to the nine families.

.

**Supplementary Figure 2.** Biplot of the principal component analysis (PCA) showing different trends in the wild emmer derivative (WED) families and their evaluated traits at Wad Medani normal sowing date (NSD) and Wad Medani late sowing date (LSD). The numbers 1 and 2 attached to the trait in the PCA indicate traits evaluated at NSD and LSD, respectively. The groups legend in the PCA indicates to the nine families. STI1-GY, stress tolerance index calculated based on the grain yield from Dongola versus that of NSD; STI2-GY, stress tolerance index calculated based on the grain yield from NSD versus that of LSD; DH, days to heading; DM, days to maturity; GFD, grain filling duration; CHLH, chlorophyll at heading; CHLM, chlorophyll at maturity; CHLD, chlorophyll degradation; GY, grain yield; BIO, biomass; TKW, thousand kernel weight; HI, harvest index; SN, seed number/spike; PHT, plant height

**Supplementary Tables**

**Supplementary Table 1**. List of the wild emmer derivative (WED) families used in the study. The recurrent parent 'Miki 3' is a durum wheat cultivar, the other nine parents (with code KU) are wild emmer wheat accessions.

**Supplementary Table 2**. Means and ranges of traits for the wild emmer derivative (WED) families evaluated at Tottori (TOT), Dongola (DON), Wad Medani normal sowing date (NSD), and Wad Medani late sowing date (LSD) during season 2019–2020.

**Supplementary Table 3**. Restricted maximum likelihood variance components analysis for 12 traits evaluated by nine wild emmer derivative families in Tottori, Dongola, Wad Medani normal sowing date and Wad Medani late sowing date.

**Supplementary Table 4**. The best top 25 lines and their families of the AMMI model, the ratio of each family in the four environments, and the top stable 25 lines across four environments from the AMMI model. Environments used to evaluate the wild emmer derivative families are Tottori, (TOT), Dongola (DON), Wad Medani normal sowing date (NSD), and Wad Medani late sowing date (LSD) during season 2019/20.

**Supplementary Table 5**. The correlation coefficient between grain yield kg ha-1 and measured traits for the wild emmer derivative families evaluated under the optimum condition at Tottori.

**Supplementary Table 6**. The correlation coefficient between grain yield kg ha-1 and measured traits for the wild emmer derivative families evaluated under the optimum condition at Dongola.

**Supplementary Table 7**. The correlation coefficient between grain yield kg ha-1 and measured traits for the wild emmer derivative families evaluated under normal sowing date (NSD) in Wad Medani.

**Supplementary Table 8**. The correlation coefficient between grain yield kg ha-1 and measured traits for the wild emmer derivative families evaluated under late sowing date (LSD) in Wad Medani.

**Supplementary Table 9**. Investigation of wild emmer wheat and recurrent parent 'Miki 3' alleles contributions to the tolerant lines in families 6, 8, and 9 by using significant markers for traits distinguish tolerant lines from susceptible ones identified under normal sowing date (NSD) or late sowing date (LSD) in Wad Medani, Sudan.
